# Differential Protein Expression in Alzheimer’s Disease Prefrontal Cortex

**DOI:** 10.1002/alz.091906

**Published:** 2025-01-03

**Authors:** Maryam Gholampour, Malay Basu, Russell H Swerdlow, Mohammad Haeri

**Affiliations:** ^1^ University of Kansas Medical Center, Kansas City, KS USA; ^2^ University of Kansas Alzheimer’s Disease Research Center, Fairway, KS USA

## Abstract

**Background:**

Understanding the proteomes of specific cell types within the brain is crucial for elucidating the mechanisms underlying Alzheimer’s disease (AD). However, the isolation and analysis of these diverse and low‐abundance cell populations remain significant challenges. This study aims to assess GeoMxTM Digital Spatial Profiler (DSP) by NanoString Technologies (NanoString, Seattle, USA) capable of spatially resolving protein expression profile of AD brains extracted from formalin‐fixed paraffin‐embedded (FFPE) specimens.

**Method:**

We analyzed protein expression profile of prefrontal cortices in post‐mortem AD brains (n = 4) and age‐matched controls (n = 4) using DSP. Regions of interest (ROIs) were targeted at gray matter and the DSP panel recorded the spatial expression of 76 proteins alongside three cell specific markers (GFAP for astrocytes, Iba1 for microglia, NeuN for neurons, and nuclear stain for DNA/nuclei). Protein expression was normalized to spiked‐in ERCC RNA controls and background‐corrected using IgG controls. Group‐wise comparisons employed linear mixed models, and pairwise comparisons used t‐tests with Benjamini‐Hochberg (BH) adjustment.

**Results:**

In each ROI, an average of 774 cells were counted, consisting of 61 GFAP, 74 microglia, and 370 neurons for each case. As expected, levels of amyloid‐β (Aβ) 1‐42, phospho‐tau (S396), and phospho‐tau (S214) were significantly elevated in AD, as confirmed by immunohistochemistry (IHC). Interestingly, Neprilysin (NEP), a key regulator of Aβ levels that catalyzes its proteolysis and prevents plaque formation, exhibited increased expression in all cell types (astrocytes, microglia, and neurons). Additionally, CD40, a crucial protein for Aβ‐induced microglial activation, showed higher levels in AD neurons, specifically.

**Conclusion:**

While there are still some limitations, DSP technology demonstrates a potential for analyzing the brain proteome in post‐mortem tissues. Screening protein expression in larger cohorts using DSP could hold promise for identifying tissue‐based biomarkers.

